# Visualisation and differentiation of binder components in hard carbon composite anodes by osmium tetroxide and uranyl acetate staining

**DOI:** 10.1111/jmi.70014

**Published:** 2025-07-21

**Authors:** Gregor Neusser, Tom Philipp, Christine Kranz

**Affiliations:** ^1^ Institute of Analytical and Bioanalytical Chemistry, Ulm University Ulm Germany

**Keywords:** FIB/SEM tomography, hard carbon composite electrode, osmium tetroxide and uranyl acetate staining, sodium carboxymethyl cellulose, styrene butadiene rubber

## Abstract

In this study, we present a protocol to visualise, track and distinguish between two different binder components commonly used for batteries, styrene butadiene rubber (SBR) and sodium carboxymethyl cellulose (CMC), within a composite hard carbon electrode for sodium‐ion batteries using a two‐step staining method. The application of osmium tetroxide (OsO_4_) vapour followed by uranyl acetate (UA) solution enables the staining of different functional groups and the individual tracing of SBR and CMC by energy dispersive X‐ray spectroscopy (EDX) measurements using the osmium (Os) and uranium (U) content. This staining procedure and the filling of the pore space with conductive platinum carbon (PtC) composite via local electron‐beam‐induced deposition (EBID) results in an excellent contrast for all components of the electrode material. The tracking and visualisation of the binder components are demonstrated with secondary electron (SE) imaging and EDX mappings at focused ion beam (FIB) prepared facets as well as with focused ion beam/scanning electron microscopy (FIB/SEM) tomography.

**LAY DESCRIPTION**: In this study, a sample preparation protocol for hard carbon (HC) composite electrode material is presented which allows to clearly distinguish between the HC particle and the two binder components, styrene butadiene rubber (SBR) and sodium carboxymethyl cellulose (CMC) in focused ion beam/scanning electron microscopy (FIB/SEM) tomography and energy dispersive X‐ray spectroscopy (EDX) measurements. For that, the material was stained with osmium tetroxide (OsO_4_) and uranyl acetate (UA) and pore space was locally filled with electron‐beam‐induced deposition (EBID) of platinum carbon (PtC).

## 1 INTRODUCTION

A clearly recognisable contrast between different phases of composite materials in scanning electron microscopy (SEM) images is a prerequisite for characterisation and quantification of certain features of interest. The lack of sufficient contrast can be a challenge, especially when different phases or components of the sample have a similar composition or density. In that case, it may be difficult to distinguish elements, as the density of the material affects the number of secondary electrons (SE) or backscattered electrons (BSE) that are able to leave the sample after interaction with the electron beam.^1^ If there is no notable variation in elemental composition and other factors like morphology or crystal orientation can be neglected, the number of detectable electrons will be similar. One way to circumnavigate this issue is the incorporation of heavy elements into the sample components, a so‐called staining or contrasting of the material.

Sample staining with heavy elements was first introduced for biological samples in the 1940s and 1950s.^2^, ^3^ Most of the established protocols for biological samples used nowadays include a preservation of the delicate ultrastructure of the cell interior by high pressure freezing, freeze substitution and fixation with the addition of osmium tetroxide (OsO_4_) and uranyl acetate (UA) solution followed by embedding of the sample in epoxy resin.^4^ OsO_4_ tends to react with non‐conjugated double bonds,^5^ whereas UA stains proteins as it reacts with carboxyl groups.^6^, ^7^ The sample preparation protocols developed for biological samples were adapted to polymer materials like rubber^5^, ^8^ and molecularly imprinted polymers.^9^ Recently, OsO_4_ staining has been applied in battery research. For example, OsO_4_ was used to stain polymeric binder components like styrene butadiene rubber (SBR) to investigate its distribution throughout the electrode composite material.^10^ Binder materials, which account for 5–10% of composite battery electrodes (anodes and cathodes) play a pivotal role for the manufacturing of battery electrodes, as they improve, e.g., their mechanical and thermal stability.^11^ Staining was also used for the successful visualisation of structural changes and degradation of a porphyrin, [5,15‐bis(ethynyl)‐10,20‐diphenylporphinato] copper(II) (CuDEPP), an active cathode material for lithium (Li)‐ion batteries material, after cycling of the battery,^12^ or investigations of Li‐ion diffusion in anodes or Li dendrites growth on the electrode surface, as OsO_4_ tends to react with Li.^13^, ^14^

In the present study, we introduce a sample preparation protocol for the visualisation of the distribution of different components of carbon‐based composite electrodes, namely a hard carbon (HC) composite electrode. HC composite electrodes are the most used anodes in rechargeable sodium‐ion batteries.^15^, ^16^ The composite electrodes contain sodium carboxymethyl cellulose (CMC) and SBR as binder mixture, HC as active material and conductive nano‐carbon particles (nano‐C) for improved conductivity of the composite. In general, the aqueous processable binders, CMC and SBR, are crucial constituents in composite battery electrodes, as they ensure good adhesion properties, high elasticity, improved cyclability and thermal stability.^17^ The uniform distribution of all compounds of composite battery electrodes, like binder, active material and conductive carbon is an important prerequisite. An inhomogeneous distribution, for example, during the fabrication and processing of the electrode, may lead to segregation, delamination or solidification which influence the overall battery performance.^18^ All parts of the composite electrode, HC, SBR, CMC and nano‐C are made mostly of carbon and therefore do not provide sufficient contrast in SEM investigations. In order to visualise all components, we applied two different staining agents to the sample, OsO_4_ to stain the double bonds of SBR and in a separate second step, UA to stain the carboxyl groups of CMC. Therefore, we were able to investigate and visualise SBR and CMC individually, which was so far only shown by using laser‐ablation laser‐induced breakdown spectroscopy (LA‐LIBS) and time‐of‐flight secondary‐ion mass spectrometry (TOF‐SIMS).^19^ The significant advantage of using staining procedure in combination with SEM compared to LA‐LIBS and TOF‐SIMS is the superior resolution by using SE, BSE and energy dispersive X‐ray spectroscopy (EDX) imaging. In this study, we used SE for imaging based on the achievable resolution compared to the BSE option on the used microscope.

**FIGURE 1:**
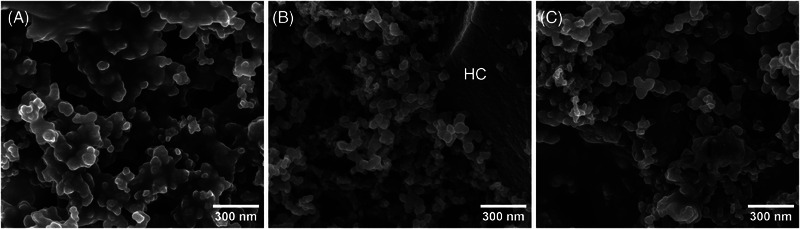
SE images of binder–nano‐C structures of HC composite electrodes with different binder composition: (A) SBR only, (B) CMC only and (C) CMC/SBR mixture 1:1. In B, a HC particle is visible.

The composite electrode contains pore space, in which SBR and CMC form delicate wormlike structures with diameters below 100 nm (Figure 1). These voids were locally filled by adopting an embedding procedure, introduced by Eswara‐Moorthy et al.,^20^ where platinum carbon (PtC) is deposited within a several micrometre deep volume using electron beam‐induced deposition (EBID). This procedure was chosen as an alternative to epoxy resin embedding, as an excellent contrast of PtC relative to HC and nano‐C as well as the stained binder due to the relatively high Pt content of PtC (15–25 atom%^21^) is obtained.

## 2 MATERIALS AND METHODS

### 2.1 HC electrode preparation and material staining

For the HC electrodes used in this study, a water‐based slurry made of 80 wt.% HC, 10 wt.% SuperP conductive carbon nano‐particles, 5 wt.% CMC and 5 wt.% SBR was prepared. An Al foil current collector was then coated with the slurry using doctor blading. The prepared electrodes were dried at ambient conditions.

Pieces of the sample with a size of 15 × 4 mm^2^ were stained in a two‐step procedure: First, samples were placed in a sealed container together with 3–4 droplets of 4% aqueous OsO_4_ solution for 6 days. The droplets were replenished after 3 days to ensure that during the whole time period sufficient staining agent was present. The droplets were not in direct contact with the sample, but the staining agent evaporates and reacts with the binder. The staining procedure was done in a fume hood due to the toxicity of OsO_4_. For the second staining step, a droplet of 2% UA dissolved in ethanol was placed directly on the sample surface for 30 min. Afterwards, the sample surface was gently rinsed with ethanol.

### 2.2 Local electron‐beam‐induced deposition (EBID) PtC embedding and facet preparation

**FIGURE 2:**
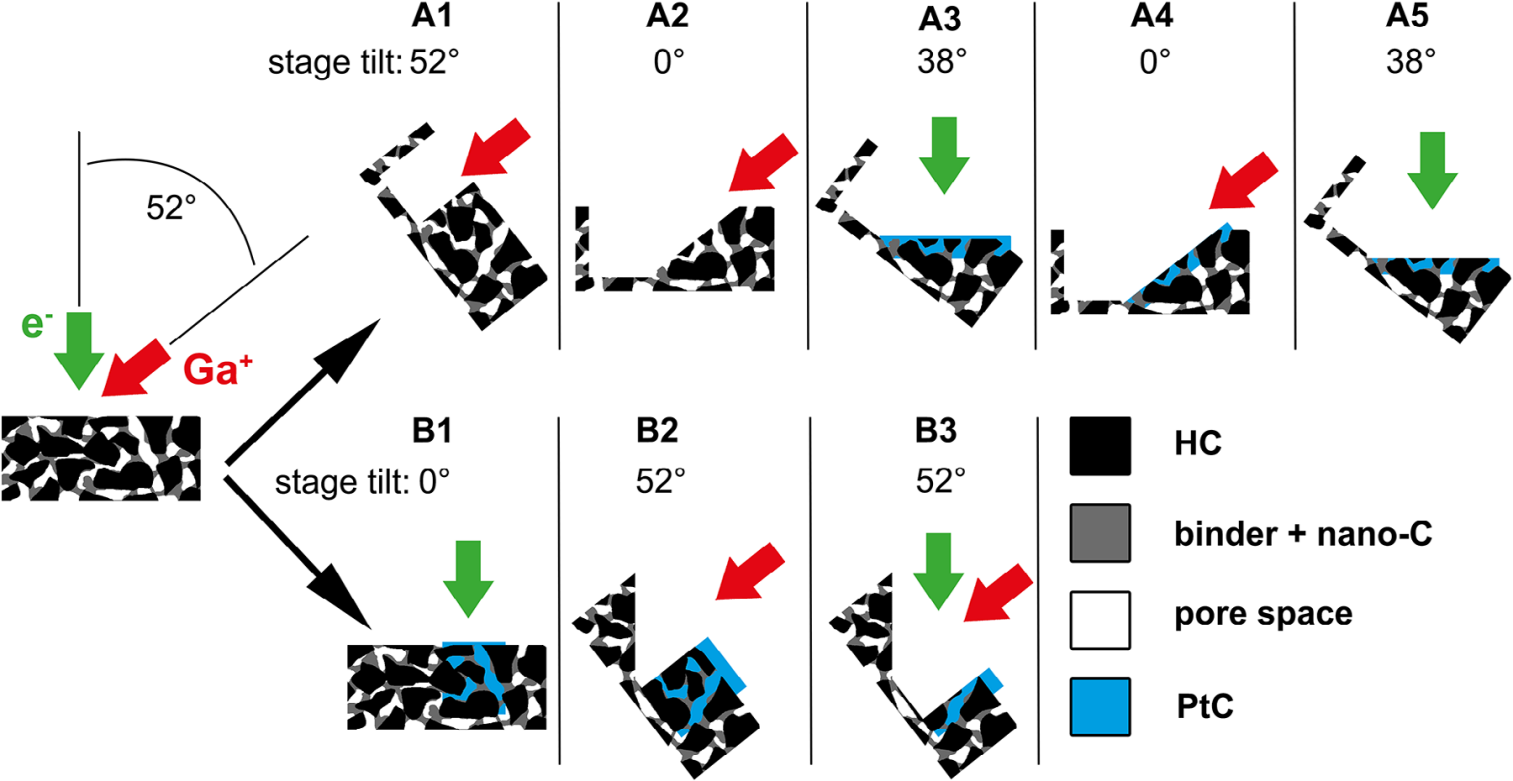
Schematic presentation of the sample preparation steps for SEM/EDX mappings (path A) and for FIB/SEM tomography (path B).

As CMC is soluble in water (but not in ethanol), the conventional embedding and mechanical polishing of the samples was avoided to ensure that the binder microstructure is not altered during sample preparation. Instead, the regions of interest of the HC composite electrode were embedded in PtC using a Quanta 3D FEG (Thermo Fisher Scientific, USA), applying two different protocols, A and B, as illustrated in Figure 2. For SEM and EDX measurements, a facet was prepared that can be investigated with the electron beam perpendicular to the prepared facet. Here, trenches in front and at the side of the region of interest were excavated to make some space for redeposition (Figure 2A1) using a focused ion beam (FIB) operated at 30 kV and 15 nA. Then a facet was prepared under a 52° angle towards the sample surface using a stage tilt of 0° (Figure 2A2) and an ion beam operated at 30 kV and 7 nA. After this step, the stage was tilted to 38° and PtC was deposited by EBID using 10 kV and 32 nA and methylcyclopentadienyl trimethyl platinum as precursor gas for the deposition (Figure 2A3). In the last step, the stage was titled back to 0° and the facet was cleaned by FIB using 30 kV and 0.5 nA (Figure 2A4). For imaging and EDX measurements, the stage was tilted again to 38° so that measurements can be done perpendicular to the surface (Figure 2A5).

For FIB/SEM tomography, a larger PtC‐embedded volume is required. Therefore, a frame of about 10 × 5 µm^2^ was irradiated with an electron beam operated at 30 kV and 32 nA again using methylcyclopentadienyl trimethyl platinum as precursor gas (Figure 2B1). Using these parameters, Pt deposition was achieved down to a depth of about 8 µm. After that, the volume for FIB/SEM tomography can be prepared as usual by FIB milling of trenches in front and beside the volume of interest and cleaning of the front facet (Figure 2B2 and B3). Additionally, a 45°‐oriented facet perpendicular to the front facet was prepared for later alignment of the FIB/SEM tomography image stack as described by Philipp et al.^12^

### 2.3 SEM imaging and EDX measurements

SE images were processed using the Fiji software package,^22^ image segmentation was done by converting the images to 8‐bit grey scale images and setting the threshold accordingly. After thresholding, possible outliers were removed using the function ‘despeckle’ of FIJI.

EDX measurements were done using an Element EDX detector (attached to the Quanta microscope) and the Apex standard software package (both EDAX Inc, Germany). Mappings were recorded using an electron beam operated at 6 kV and 16 nA with a dwell time of 50 µs for 64 frames. The mapping resolution was 1024 × 800 pixel with a pixel size of approximately 7 × 7 nm^2^. EDX spectra were measured at 6 kV and 8 nA using a dwell time of 60 s.

FIB/SEM tomography was performed using a Helios Nanolab 600 (Thermo Fisher Scientific, USA) and the Auto Slice and View G3 software package (Thermo Fisher Scientific, USA). An electron beam operated at 3 kV and 86 pA was used and the SE images were recorded with the immersion mode (SE mode). The image pixel size was 5 × 5 nm^2^ with a slice thickness of 15 nm. A set of 72 images was chosen from the whole data set and aligned, cropped and segmented manually using Avizo 3D software package (Thermo Fisher Scientific, USA).

## 3 RESULTS AND DISCUSSION

### 3.1 EBID embedding

**FIGURE 3:**
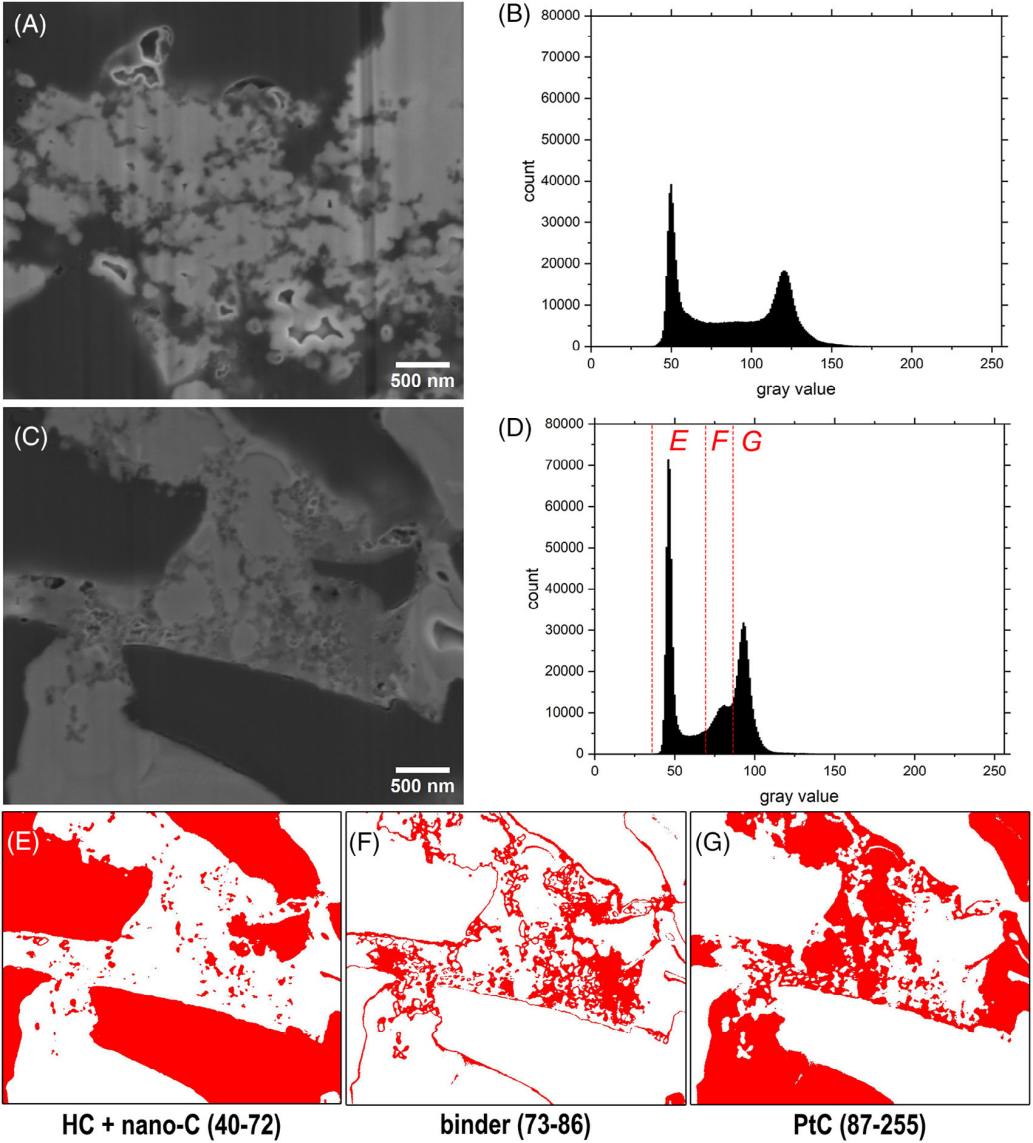
SE images of cross‐sections of PtC‐embedded HC composite electrodes with the corresponding histograms of the pixel values. The cross‐sections were prepared following the method for path A (Figure 2). (A) The non‐stained sample, (C) the OsO_4_ and UA‐stained sample. (B, D) The histograms of the SE images shown in A and C, respectively. Both images shown in A and C were recorded using the same imaging parameters (5 kV, 23 pA, horizontal field width of 4.26 µm, dwell time of 100 µs per pixel and same contrast and brightness adjustments). (E–G) The resulting images from applying the thresholds to the image shown in C. (E) The pixel with grey values ranging from 40–72, (F) in the range of 73–86 and (G) in the range of 87–255, respectively. The threshold regions are depicted in D with red dashed lines separating the areas used for E, F, and G, respectively.

**FIGURE 4:**
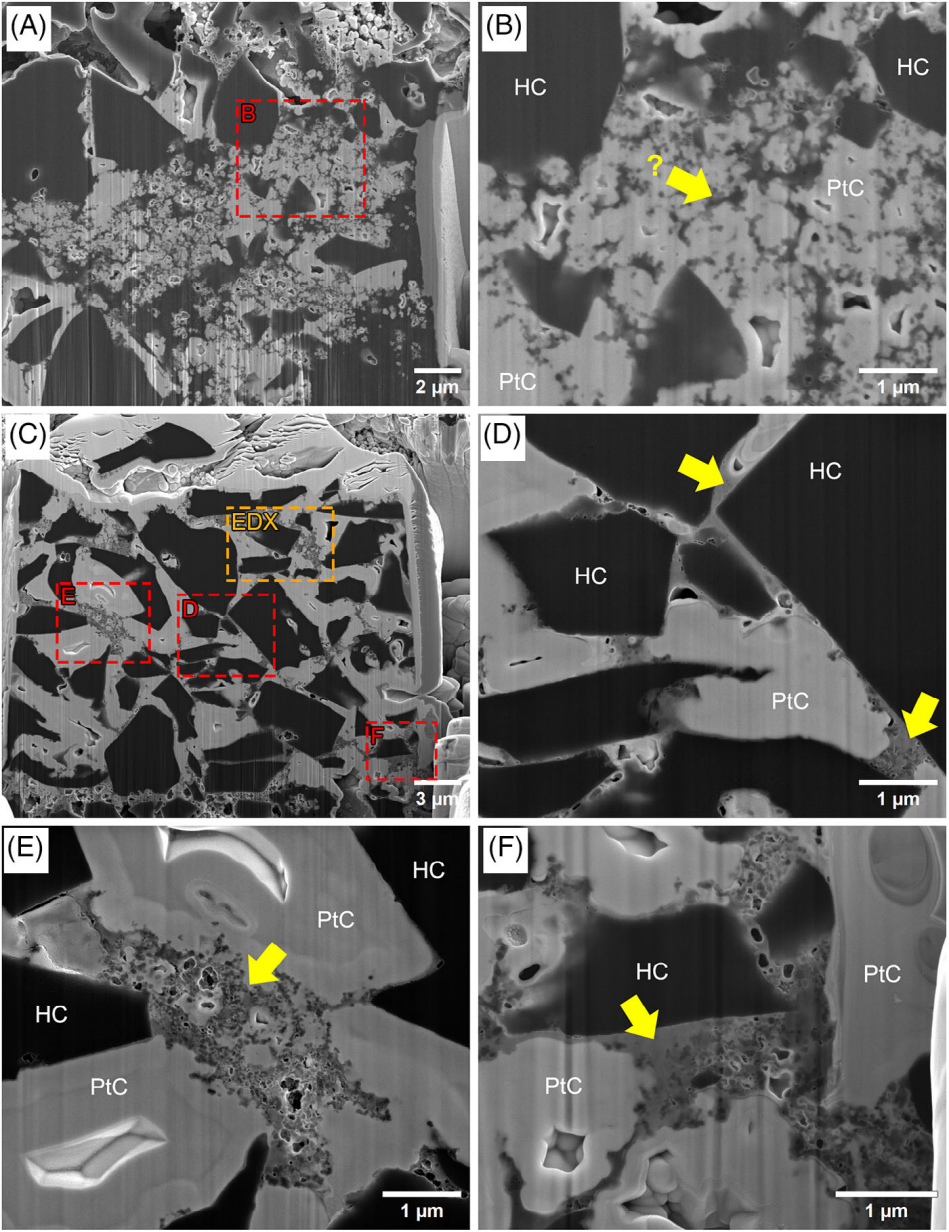
SE images of cross‐sections of PtC‐embedded HC composite electrodes. The cross‐sections were prepared following the method for path A (Figure 2). (A, B) A non‐stained sample. (C–F) The OsO_4_ and UA‐stained sample. The yellow arrows point to regions containing binder material. The red boxes in A and C mark the areas of the zoomed‐in images shown in B and D–F, respectively. The orange box in C marks the area which was used for the EDX mapping that is shown in Figure 6.

The HC composite electrode material is highly porous with pore sizes in the range of approximately 100 nm up to several µm in diameter (Figures 3 and 4). Therefore, it is a prerequisite to fill the pore space prior to facet preparation and SEM imaging, mainly for two reasons: First, the occurrence of voids causes curtaining effects,^23^ vertically oriented stripes of thin depressions and ridges within the facet surface. This pattern of stripes superimposes features of the prepared facet especially in SE images and may hinder a correct interpretation of structures visible within the image. Secondly, the occurrence of voids may change the brightness of pixel at the edge of the voids due to the edge effect.^1^ Another problem can arise from voids present in a tomogram as the back of the void is present on several consecutive images. These so‐called shine‐through artefacts can complicate segmentation of the image stack and lead to erroneous interpretation of the data.

For the presented studies, local embedding of the electrode material by EBID‐induced PtC deposition was chosen. A significant advantage of using PtC for embedding is that this step is less time consuming than the conventional embedding procedure of the entire sample in an epoxy resin followed by mechanical grinding and polishing to achieve a flat sample surface. Local EBID embedding can be completed in a couple of hours, whereas resin embedding and polishing procedure typically takes days. As the CMC binder is soluble in water, another important advantage of EBID embedding is that contact with water is avoided. Therefore, the delicate structure of the binder/nano‐C mixture within the composite is not altered during the sample preparation.

Local EBID embedding fills most of the pore space within the area of interest, depending on the initial size of the pore; up to 30% open space remains in the case of the largest pores of about 3 µm in diameter. Nonetheless, shine‐through artefacts and curtaining effects during FIB facet preparation are significantly reduced. Small voids remain open within the stained binder/nano‐C mixture (visible in the SE images shown in Figure 4). This may occur when PtC is deposited at the surface of a pore, probably blocking the precursor gas to enter the now encapsulated void. Furthermore, as the PtC layer grows, more electrons scatter and lose all their energy without entering the void. As a result, such isolated voids cannot be completely filled with PtC. The high density of PtC, due to its Pt content of 15–25 atom%,^21^ which prevents the complete filling of the pore space, is on the other hand also an advantage as it also provides high contrast relative to the stained binder components, as well as the HC particle and nano‐C, which would not be the case if epoxy resin were used instead (Figures 3 and 4). Due to its relatively high density, PtC also improves the resolution of the EDX measurements by reducing the extent of the excitation volume.

### 3.2 Contrast enhancement via binder staining

Without staining of the electrode sample, HC particles, nano‐C and the used binders SBR and CMC exhibit similar grey values in SE images and contrast is only observed between the PtC filled pore space and the material mix of the three components (Figures 3A and 4A and B). As a consequence, the histogram of the SEM image in Figure 3B shows a bimodal distribution of grey values with a strong peak around 50 representing the HC particles, binder and nano‐C, and a peak around 120 for the PtC‐filled pores, respectively.

After staining the samples with OsO_4_ and UA, a contrast change between the carbon materials (HC particles and nano‐C), which reflect the darkest grey values and the binder components (mid grey), and the PtC‐filled pores (brightest) was obtained. Therefore, the different components of the electrode are easily distinguishable (Figures 3C and 4C–F). By staining the binder components, the nano‐C particles can be located within the binder matrix (Figure 4E and F). The histogram of SEM images now shows a multimodal distribution of grey values; in the case of the SE image shown in Figure 3C, three peaks can be identified, one around 46 for HC particles and the nano‐C, one at 93 for the PtC, and one small peak between these two around 81 for the binder components.

The peaks identified in Figure 3D can be used for thresholding and therefore segmentation of SE images. This was done exemplary for the SE image shown in Figure 3C, here a threshold in the range of 40–72 was applied to mark the pixel for HC and nano‐C, the range between 73–86 was used for the binder, and the range 87–255 was used to mark all pixels that belong to the PtC‐filled pores (Figure 3E–G). Overall, this segmentation is sufficient, except the border regions between the HC particle and the PtC, as it seems unlikely that the entire HC particle surface is covered with a thin layer of binder, as implied in the binarised image shown Figure 3F. All SEM images used are SE images, but it is important to point out that the SE signal is affected by the number of BSE leaving the sample as those producing SE on the way.^1^ As the BSE originates from a volume, measured pixel located directly on top or next to the border between HC and PtC may have BSE originating from both phases. Pixels around borders of material with different densities, that is, light and dense material may have an intermediate grey value in SE images. Therefore, the intermediate grey values observed in such border regions may not be associated with the presence of binder material.

### 3.3 FIB/SEM tomography study

**FIGURE 5:**
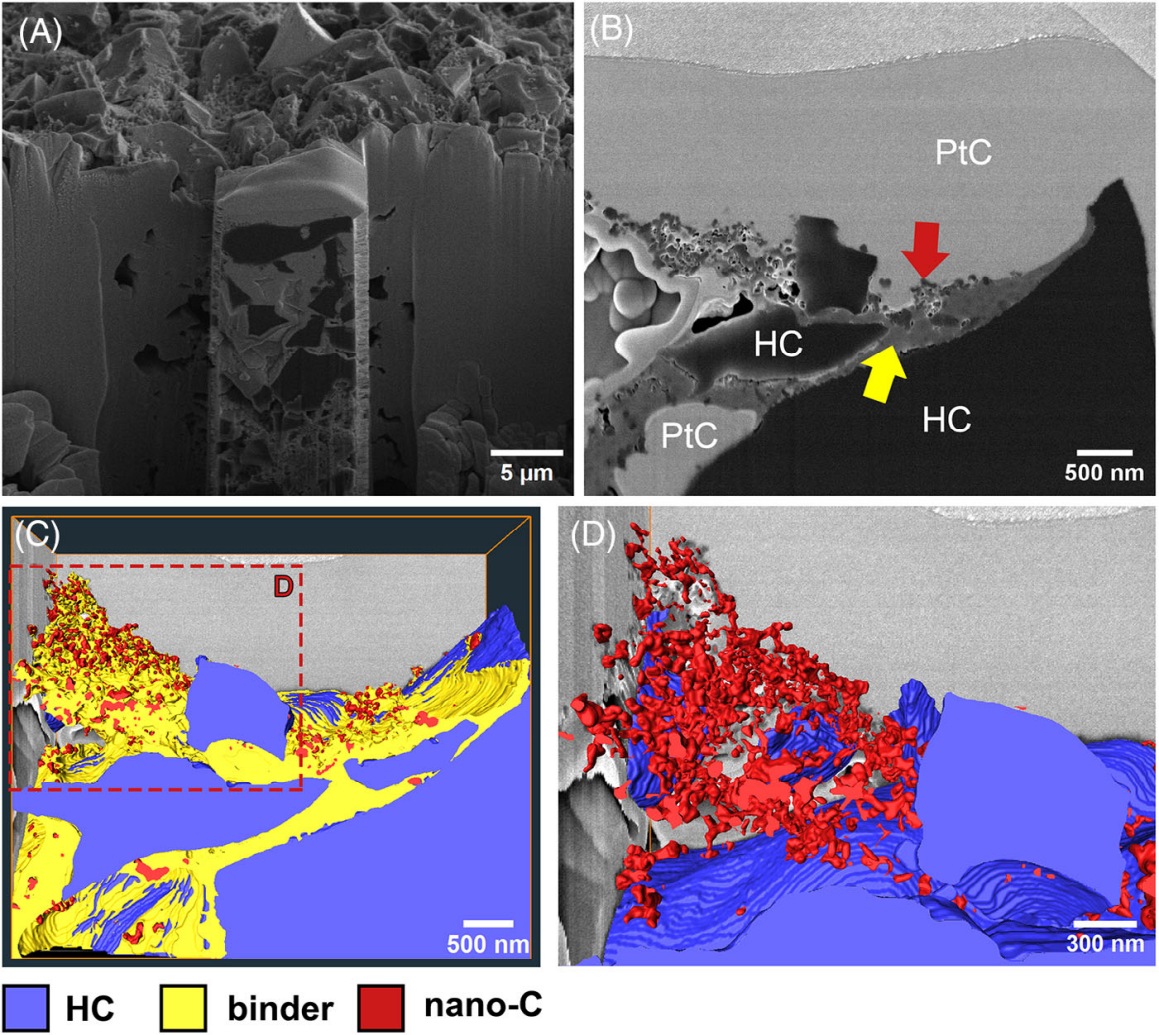
(A) A SE image of the prepared volume of the sample prior to FIB/SEM tomography. (B) A SE image from the tomography data set used for segmentation. The 3D visualisation is depicted in C and D, respectively. Colour code is the same as for the arrows in B and segmented parts shown in C and D. In D, the binder is not shown to provide a clearer view of the nano‐C distribution. The red box in C marks the area of the zoomed in area shown in D.

The SE images of the CMC–SBR agglomerates shown in Figure 1 indicate that the binders form three‐dimensional structures within the pore space. In order to clarify the relationship of observed structures relative to the HC particle surface, FIB/SEM tomography of a volume of 5.12 × 4.42 × 1.08 µm^3^ was performed. The excellent contrast in the SE images provided by the combination of staining of both CMC and SBR and local EBID PtC embedding allows an identification of the nano‐C particle distributed within the binder matrix (Figure 5B), which would not be possible without it. As it turns out, most of the nano‐C particles revealed by FIB/SEM tomography are located within the binder matrix (Figure 5C and D), without any connection to the HC particle surface. Nano‐C particles are added to the electrode mixture to enhance electric conductivity, their distribution among the electrode material plays an important role for the overall performance of the battery material.^18^, ^24^ It turns out that for the electrode investigated in this study, most of the nano‐C content forms agglomerates with the binder and is isolated from the HC particle surface. This issue may be addressed by adjusting the amount of binder and/or nano‐C used for the manufacturing of the composite electrode. Usually for HC composite electrodes, a binder content of 5–10 wt% is used,^17^ but the total amount of binder used in this study, 10 wt%, may be too high.

Although the contrast provided is sufficient to use thresholding for fast segmentation, segmentation of border areas between HC particle and PtC with intermediate grey values must be carefully checked as stated earlier. As the local EBID PtC embedding still leaves pore space open, shine through and edge effects may still be of an issue. In the segmentation shown here, the verification of the segmented images was done manually as the dataset contains only 72 images. In the case of large datasets, the use of automatic segmentation algorithms or machine learning tools like the trainable WEKA segmentation provided for Fiji software package^25^ may be attractive to limit the amount of time used for segmentation and verification.

### 3.4 Individual tracking of SBR and CMC binder components by EDX

**FIGURE 6:**
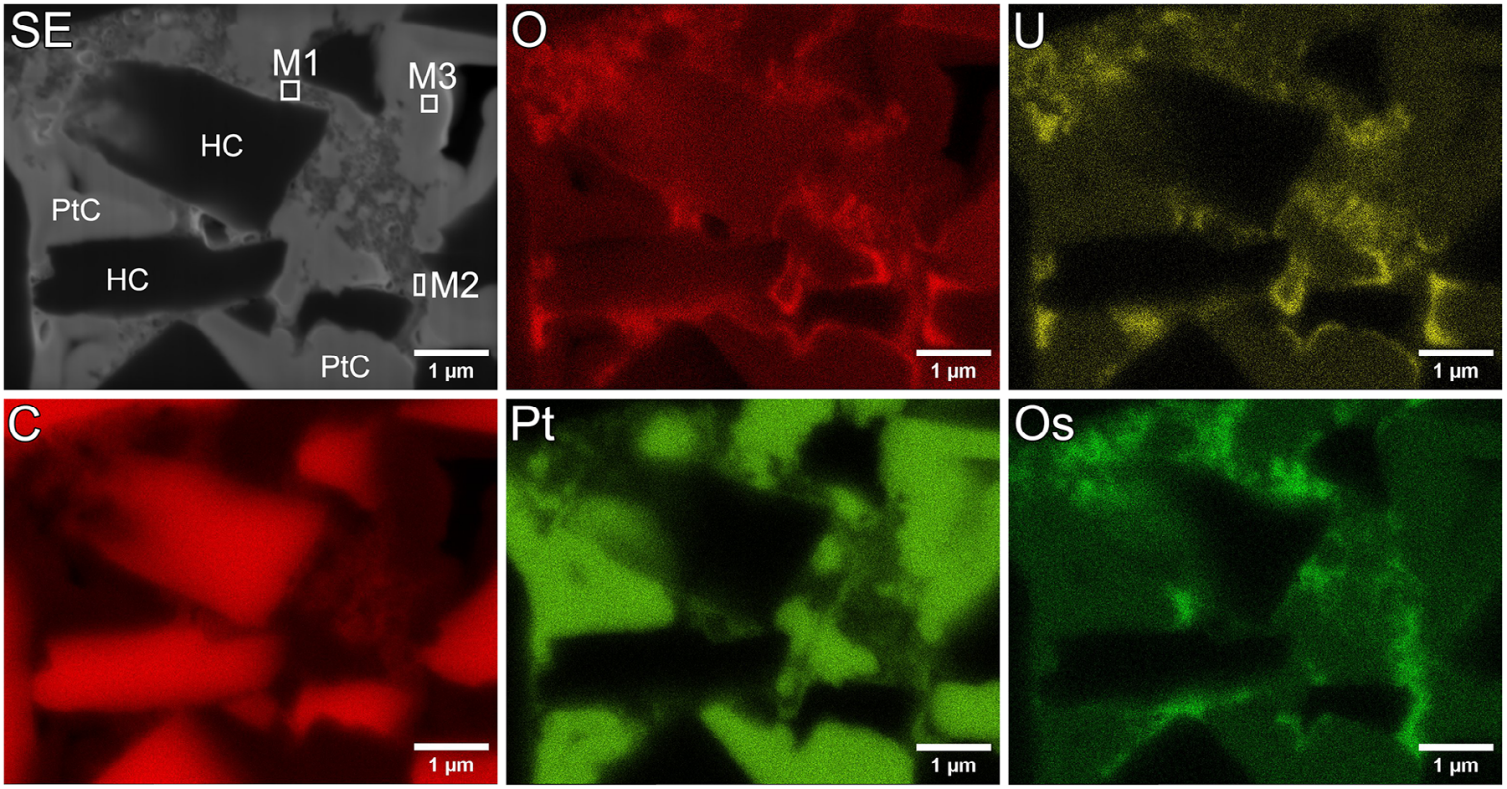
EDX mapping showing the elemental distribution of C, Pt, O, U, Os as well as a SE image of the mapped area. The areas M1–M3 marked in the SE images show the areas where EDX spectra were collected in a separate measurement; these spectra are shown in Figure 7B.

**FIGURE 7:**
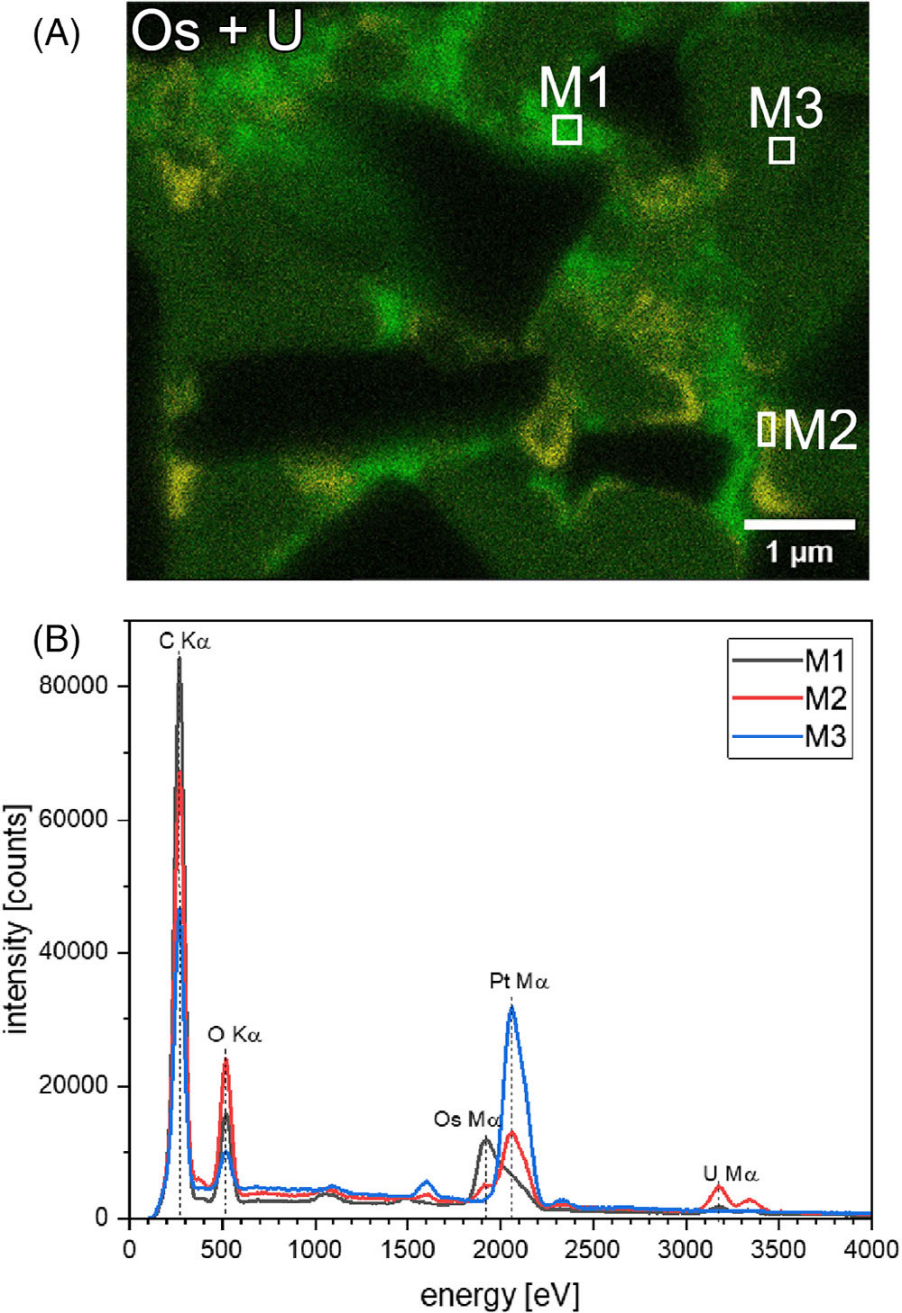
(A) Overlay of the Os (green) and U (yellow) elemental distribution maps shown in Figure 6. (B) Plot of EDX spectra of the selected areas M1–M3 marked in A and Figure 6.

In the sections above, we demonstrate the improved contrast in SEM images and therefore the possibility to distinguish between HC, nano‐C, PtC filled pores and the binder, as a result of staining. As two different staining agents were used, OsO_4_ and UA, and as stated above, OsO_4_ reacts with non‐conjugated double bonds and therefore only with SBR^10^ whereas UA reacts with carboxyl groups of CMC,^7^ the two binder types can be distinguished in EDX mappings due to the Os and U content. This is shown in the EDX elemental distribution maps shown in Figure 6 and for better clarity regarding Os and U distribution in the overlay in Figure 7A. The maps of Oxygen (O), U and Os reveal the distribution of the binder mixture, SBR coincides with the Os distribution, CMC with U and O coincides with both binder types. SBR and CMC are separated and are not appearing as a mixed phase, as Os and U are not in the same area. The maps of C and Pt coincide with the location of the HC particle and the filled pore space, respectively.

The separation of binder components was confirmed by comparing EDX spectra of an area enriched with Os (Figures 6 and 7A region M1) with the spectra taken from an area enriched in U (Figures 6 and 7A region M2). The spectra of the marked areas are shown in Figure 7B. They clearly show that either Os or U is present in the measurement. The spectra of an area containing only PtC (Figures 6 and 7A region M3) was taken to verify that the Os Mα line (1.910 keV) and the Pt Mα line (2.048 keV) can be clearly distinguished in the EDX measurements.

The possibility to individually track the different binder components by EDX mappings provides important information. For instance, information on migration of the binder components during the drying process of the slurry or segregation during long‐term cycling can be obtained with high resolution.

## 4 CONCLUSION

In this study, we explore the potential of sample preparation including staining for the visualisation of HC composite electrode components, with HC particles as active material, nano‐C particles for improved conductivity and a binder mixture of CMC and SBR for adhesion and cohesion. The sample preparation protocol consists of a two‐step staining, local EBID‐induced PtC embedding and facet preparation by FIB milling that may also be applied to other battery materials, where the binder and nano‐C distribution plays a pivotal role. The two‐step staining allows for the contrasting of the binder mixture relative to the HC and nano‐C particles, as well as providing the possibility to reveal the distribution of SBR and CMC individually by EDX measurements. We could demonstrate that the distribution of the binders can be visualised with EDX mappings due to local enrichment of Os and U, respectively. Another future application is the investigation of battery electrode material after cycling. If functional groups within the binder components, namely carboxyl groups in the case of CMC and unconjugated double bonds in the case of SBR, are altered, the binder components are not able to react with the staining agents anymore. A comparison with pristine material will shed some light on if and to what extent the binder material is altered during cycling of the battery material or whether segregation within the composite material occurs. This insight may help to understand alteration of battery materials during its life cycle and provide knowledge for further optimisation of electrode materials, for example, for post‐Li batteries.
